# Developing an RAI-MDS Behavior-Based Pain Scale for Long-Term Care Residents With Advanced Dementia

**DOI:** 10.1093/geroni/igaa057.658

**Published:** 2020-12-16

**Authors:** Jennifer Knopp-Sihota, Matthias Hoben, Jeff Poss, Carole Estabrooks

**Affiliations:** 1 Athabasca University, Edmonton, Alberta, Canada; 2 University of Alberta, Edmonton, Alberta, Canada; 3 University of Waterloo, Vancouver, British Columbia, Canada

## Abstract

In Canadian and many international long-term care (LTC) facilities, pain assessment frequently relies on data from the Resident Assessment Instrument – Minimum Data Set 2.0 (RAI-MDS). The RAI-MDS produces a two-item scale, measuring both pain frequency and pain intensity. This scale correlates well with self-reported pain in cognitively intact LTC residents, but despite repeated testing, is less valid for use in residents with more advanced cognitive impairment who are unable to self-report their pain. In this study we aimed to develop and validate a behaviour-based pain assessment scale for long-term care residents using data available in the RAI-MDS. To construct our initial scale, we reviewed the literature and compiled a list of observable indicators of pain (e.g., grimacing) and linked these with 28 similar items available in the RAI-MDS. Using Delphi techniques, we further refined this to 20 items. We then evaluated the psychometric properties of our scale using two independent, representative samples, of urban LTC residents in Western Canada. Exploratory factor analyses were conducted in sample one (n=16,282) and confirmatory factor analyses (CFA) were then conducted in sample two (n=15,785) in order to test, and confirm, our model. A two-factor solution was identified grouping RAI-MDS items into subscales 1) change in status (e.g., new onset restlessness) and 2) behaviours (e.g., crying). Commonly recognized model fit indices were acceptable suggesting the adequacy of the two-factor solution. Results provide preliminary support for the use of behavioural-based pain assessment scale using RAI-MDS data. Further evaluation and validation of our scale is warranted.

